# Comparative genomic analyses of Polymyxin-resistant *Enterobacteriaceae* strains from China

**DOI:** 10.1186/s12864-022-08301-5

**Published:** 2022-01-31

**Authors:** Zhien He, Yongqiang Yang, Wei Li, Xiaoling Ma, Changfeng Zhang, Jingxiang Zhang, Baolin Sun, Tao Ding, Guo-bao Tian

**Affiliations:** 1 Department of Oncology, The First Affiliated Hospital, University of Science and Technology of China, Hefei, China; 2 Department of Microbiology, Zhongshan School of Medicine, Sun Yat-sen University, Guangzhou, 510080 China; 3 Key Laboratory of Tropical Diseases Control (Sun Yat-sen University), Ministry of Education, Guangzhou, 510080 China; 4 School of Pharmaceutical Sciences (Shenzhen), Sun Yat-sen University, Guangzhou, 510006 China; 5 Clinical Laboratory of the First Affiliated Hospital, Anhui University of Chinese Medicine, Hefei, 230031 Anhui China; 6 Department of Immunology, Zhongshan School of Medicine, Sun Yat-sen University, Guangzhou, 510080 China; 7 Xizang Minzu University School of Medicine, Xianyang, China

**Keywords:** *Mcr-1*, *Klebsiella pneumoniae*, Antibiotic resistance, Comparative genomic

## Abstract

**Background:**

Mobile colistin resistance like gene (*mcr*-like gene) is a new type of polymyxin resistance gene that can be horizontally transferred in the *Enterobacteriaceae*. This has brought great challenges to the treatment of multidrug-resistant *Escherichia coli* and *K. pneumoniae*.

**Results:**

*K. pneumoniae* 16BU137 and *E. coli* 17MR471 were isolated from the bus and subway handrails in Guangzhou, China. *K. pneumoniae* 19PDR22 and KP20191015 were isolated from patients with urinary tract infection and severe pneumonia in Anhui, China. Sequence analysis indicated that the *mcr-1.1* gene was present on the chromosome of *E. coli* 17MR471, and the gene was in the gene cassette containing *pap2* and two copies of IS*Apl1*.The *mcr-1.1* was found in the putative IncX4 type plasmid p16BU137_mcr-1.1 of *K. pneumoniae* 16BU137, but IS*Apl1* was not found in its flanking sequence. *Mcr-8* variants were found in the putative IncFIB/ IncFII plasmid pKP20191015_mcr-8 of *K. pneumoniae* KP20191015 and flanked by IS*Ecl1* and IS*Kpn26*.

**Conclusion:**

This study provides timely information on *Enterobacteriaceae* bacteria carrying *mcr*-like genes, and provides a reference for studying the spread of *mcr-1* in China and globally.

**Supplementary Information:**

The online version contains supplementary material available at 10.1186/s12864-022-08301-5.

## Introduction

Polymyxin is a cyclic lipopeptide antibiotic discovered by Ainsworth et al. [1] in the 1940s. In 1959 [2], polymyxin B and colistin (polymyxin E) were introduced into clinical practice and used to treat infections caused by gram-negative bacteria. Due to the strong nephrotoxicity and neurotoxicity, and the popularity of more “safe” antibiotics such as beta-lactam antibiotics, polymyxins had not been used in clinical treatments in the following decades. In the past two decades, the outbreak of multidrug resistant (MDR) gram-negative bacteria and the lack of new antibiotics have caused polymyxins to return to clinical application as the last line of defense against gram-negative bacteria [3].

The resistance mechanisms of bacteria to polymyxins are mainly divided into two categories, two-component system [4, 5] and hyperproduction of CPS capsular polysaccharide (CPS) [6]. The two-component system mainly regulates polymyxin resistance by PhoPQ and PmrAB in *Enterobacteriaceae*, such as *Pseudomonas aeruginosa* and *Salmonella enterica* server Typhimurium. PhoQ can phosphorylate and activate PhoP in the presence of polymyxin. PhoP can increase the positive charge of the outer membrane of the bacteria and the resistance to polymyxins by activating the *pmrHFIJKLM* operon, causing lipid A to be modified by 4-amino-4-arabinose. Hyperproduction of CPS generally occurs in *K. pneumoniae*. Some *K. pneumoniae* strains can reduce the interaction between polymyxin and bacterial surface by synthesizing large amounts of CPS, which leading to the development of polymyxin resistance. Efflux pumps of some Gram-negative bacteria (such as AcrAB [7] and KpnEF [8] of *K. pneumoniae*) can participate in the resistance of bacteria to polymyxins, but the molecular mechanism is not yet clear. Although bacteria have evolved multiple polymyxin-resistance mechanisms, these mechanisms often require sacrificing their own development and are difficult to disseminate horizontally between strains. These factors limit the spread of these resistant genes among strains. However, in 2015, China reported a new colistin resistance gene, *mcr-1*, carried by *E. coli* in the intestine of edible pigs, can be transferred horizontally in *Enterobacteriaceae* [9]. According to statistics before 2016, *mcr-1* positive strains have been reported in more than 40 countries [10], spreading across 7 continents, and may be further expanded. Several reports have shown that many drug-resistant genes, such as New Delhi β-lactamase (NDM) and other extended spectrum β-lactamase genes (ESBLs), were frequently found in the strains carrying *mcr-1* [11, 12]. The emergence of *mcr-1* not only subverted our understanding of polymyxin resistance genes, but also greatly increased the difficulty of treating MDR pathogenic microorganisms.

MCR-1 is a phosphoethanolamine (PEA) transferase with a 5-fold hydrophobic transmembrane helix located in the periplasmic domain and can reduce the net negative charge of the outer membrane of the bacteria by modifying PEA on the negatively charged lipid A on the lipopolysaccharide (LPS) of the bacteria [13]. The modification reduces the interaction of polymyxin on the outer membrane of bacteria, which in turn produces resistance to polymyxin [14]. Generally, *mcr-1* forms a complex transposon *Tn 6330* with the surrounding transposon sequence IS*Apl1* [15]. The complex transposon consists of a sequence of about 2600 bp containing *mcr-1* (1626 bp), a PAP2 superfamily protein encoding gene (765 bp), and IS*Apl1* transposon insertions on both sides [16]. IS*Apl1* belongs to the IS*30* family and therefore has similar functions and activities to IS*30* members [17]. It is flanked by 27 bp inverted repeats (referred to as *IRL* and *IRR*) and contains a 927 bp open reading frame (*orf*). The IS*Apl1* transposon will self-cleave to form a circular sequence intermediate (IS*Apl1*)_2_-*mcr-1*-*pap2* [18, 19] if the IS*Apl1* transposon exists around the *mcr-1* gene. The circular intermediate contains 2 bp of host flanking DNA between adjacent IS*Apl1* transposon ends and generates 2 bp of target site duplications (TSDs) after integration [20]. When the *mcr-1* circular intermediate is integrated into the plasmid or genome of another strain, there is a probability that the IS*Apl1* transposon sequence will be lost. Loss of IS*Apl1* stabilizes *mcr-1* in the plasmid or genome, which is conducive to the widespread spread of *mcr-1*.

Epidemiological studies have found that *mcr-1* can be horizontally transferred in more than a dozen *Enterobacteriaceae*, mainly including *E. coli*, *K. pneumoniae*, *Salmonella* spp. [21], *Enterobacter aerogenes* [22], *P. aeruginosa* [23], *Proteus putida* [24], *Enterobacter cloacae* [22], *Cronobacter sakazakii* [25], *Shigella sonnei* [26], *Kluyvera ascorbate* [27], *Raoultella ornithinolytica* [28], *Achromobacter spp* [23] and *Citrobacter spp* [29]. These bacteria are mainly transmitted in nature through soil, water, food chains and animal migration [30, 31], and further lead to the global spread of *mcr-1*. The whole genome sequencing results of *mcr-1* positive strains showed that the *mcr-1*-bearing plasmids were mainly IncI2, IncX4, IncHI2 [32], IncP [33], IncHI1 [34], IncFI, IncFII [35], IncFIB [36], IncK [37], IncY [38], IncN [31], F18:A–:B+ [39]. Among them, IncI2, IncX4 and IncHI2 are the main replicons, and are all conjugative transfer plasmids. These carried plasmids can be stably present in the recipient bacteria even in the absence of polymyxin.

Since *mcr-1* was discovered, not only twenty-five genetic variants of the *mcr-1* gene (such as *mcr-1.1*, *mcr-1.2*, etc.) were reported all over the world [40, 41], but also a variety of *mcr*-like genes were discovered, which were named *mcr-1*, *mcr-2* [42], *mcr-3* [43], *mcr-4* [44], *mcr-5* [45], *mcr-6* [46], *mcr-7* [47], *mcr-8* [48], *mcr-9* [49] and *mcr-10* [50]. Among them, *mcr-2* and *mcr-3* were found in *E. coli*. *Mcr-4*, *mcr-5* and *mcr-9* were found in *S. enterica subsp*. The *mcr-7* and *mcr-8* were found in *K. pneumoniae*. The *mcr-6* was found in *Moraxella* spp. These proteins encoded by these *mcr*-like genes have different amino acid sequence identity with MCR-1. MCR-6 has the highest amino acid sequence similarity to MCR-1 (82.7%), while MCR-4 has the lowest amino acid sequence similarity to MCR-1 (32.1%), so their sources are not the same. Among them, MCR-1 and MCR-2 are similar in structure, and there are PAP2 family protein coding genes downstream of the coding genes, and the transposition element located near *mcr-2* is IS*1595* instead of IS*Apl1*. The structures of MCR-3, MCR-4 and MCR-9 are similar. In addition, Teo et al. [51], showed that the coexistence of some *mcr*-like genes did not significantly improve the polymyxin resistance of clinical *Enterobacteriaceae* strains.

In the current study, we performed a third-generation genome sequencing analysis of three strains of polymyxin B resistant *K. pneumoniae* (16BU137, KP20191015 and 19PDR22) and one strain of polymyxin B resistant *E. coli* (17MR471) from patients and environment. Then we combined with the phenotypes of related experiments to explain the resistance mechanism of *mcr*-like genes.

## Results

### Four multidrug-resistant strains all showed colistin resistance

**Fig. 1:**
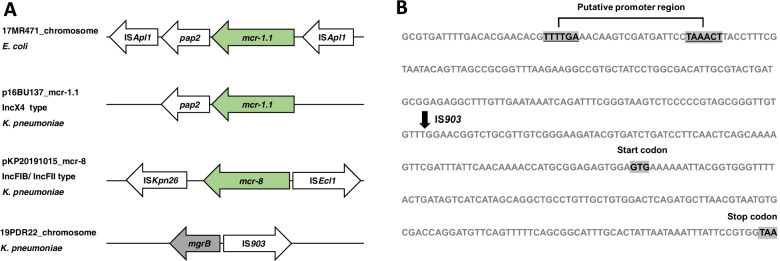
The genetic environment of *mcr*-like genes and *mgrB*. **A** The genome of *E. coli* 17MR471 contains the *mcr-1.1* gene. The IncX4 type plasmid p16BU137_mcr-1.1 of *K. pneumoniae* 16BU137 contains the *mcr-1.1* gene. The IncFIB/ IncFII type plasmid pKP20191015_mcr-8 of *K. pneumoniae* KP20191015 contains the *mcr-8* variant. No *mcr*-like gene was detected in *K. pneumoniae* 19PDR22. **B** The upstream sequence of *mgrB* in 19PDR22 was inserted by IS*903*. The arrow box indicate the target site for insertion of IS*903*

We obtained four MDR strains resistant to polymyxin B, including three strains of *K. pneumoniae* (16BU137, KP20191015 and 19PDR22) and one strain of *E. coli* (17MR471). According to the whole-genome three-generation sequencing results, *E. coli* 17MR471 and *K. pneumoniae* 16BU137 carried the *mcr-1.1* genes, and *K. pneumoniae* KP20191015 carried the *mcr-8.2* gene (Fig. 1A). To determine the phenotypes of these four strains, we performed the determination of MIC value (Table 1). Based on polymyxin B resistance criteria (USCAST, MICs, ≥4 μg/ml) [53], these strains were identified as polymyxin B resistant strains. The MIC values of the four strains were 4 μg/ml (*E. coli* 17MR471), 8 μg/ml (*K. pneumoniae* 16BU137), 32 μg/ml (*K. pneumoniae* KP20191015) and 64 μg/ml (*K. pneumoniae* 19PDR22). Among these strains, *K. pneumoniae* 19PDR22 has the highest MIC value and *E. coli* 17MR471 has the lowest MIC value.

**Table 1:** Strains used in this study

Strain	Source^a^	MIC of polymyxin B (μg/ml)	MLST	Data^b^	Region^c^
Strains					
16BU137	Bus handrail	8	37	2016.12.17	Guangdong, China
17MR471	Subway handrail	4	1437	2017.10.28	Guangdong, China
KP20191015	Sputum	32	340	2019.08.02	Anhui, China
19PDR22	Urine	64	11	2019.09.16	Anhui, China
J53 [52]	Laboratory	0.5	10	2018.05.24	Kyoto, Japan
J53-16BU137	Laboratory	4	10	2021.01.02	Anhui, China

^*a*^Source, source of isolates

^*b*^Data, date of isolate collection

^c^Region, geographic location of isolate collection

### Transferability of *mcr-1*- and *mcr-8*-carring plasmids

Transconjugants conjugate of J53 and 16BU137 is called J53-16BU137, and transconjugants of J53 and KP20191015 is called J53-KP20191015. Through the drug susceptibility test, we found that neither 16BU137 nor KP20191015 can grow on MH plates containing 100 mg/L of sodium azide. PCR detection of *mcr*-like gene and *K. pneumoniae*-specific gene was performed on all transconjugants. Using *wzi* gene as a *K. pneumoniae*-specific gene (Table 2). PCR product of J53 showed no bands in agarose gel electrophoresis, showing negative, and other *K. pneumoniae* showed positive. The PCR result of *mcr-1* of J53-16BU137 was positive, and the PCR result of *wzi* was negative, indicating that the *mcr-1* plasmid carried by 16BU137 was successfully transferred to the J53, and the conjugation efficiency was 1 × 10^− 4^ per donor cell. The MIC of J53 for polymyxin B is 0.5 μg/ml, and the MIC of J53-16BU137 for polymyxin B is 4 μg/ml. It is proved that J53 is transformed from a polymyxin-sensitive strain into a polymyxin-resistant strain after receiving p16BU137_mcr-1.1. Although we also performed the same conjugation experiment on KP20191015, we did not observe the transfer of the plasmid carrying *mcr-8* from KP20191015 to J53, which may indicate that the *mcr-8* plasmid carried by KP20191015 is difficult to transfer between different strains.

**Table 2:** Sequences of primers used in this study

Primer	Oligonucleotide (5′-3′)^a^	Application
*mcr-1* -F	GTCAGTCCGTTTGTTCTTG	Detection
*mcr-1* -R	GGTGACATCAAACAGCTT	Detection
*mcr-8* -F	CAACATAGCACTTTGGCA	Detection
*mcr-8* -R	GGAAGACAGTGGTGTGTG	Detection
*wzi*-F	ATGATAAAAATTGCGCGCAT	Detection
*wzi*-R	GCGTGATCCGTTGCTGATCC	Detection

### Genomic profiles of four colistin-resistant isolates

**Fig. 2:**
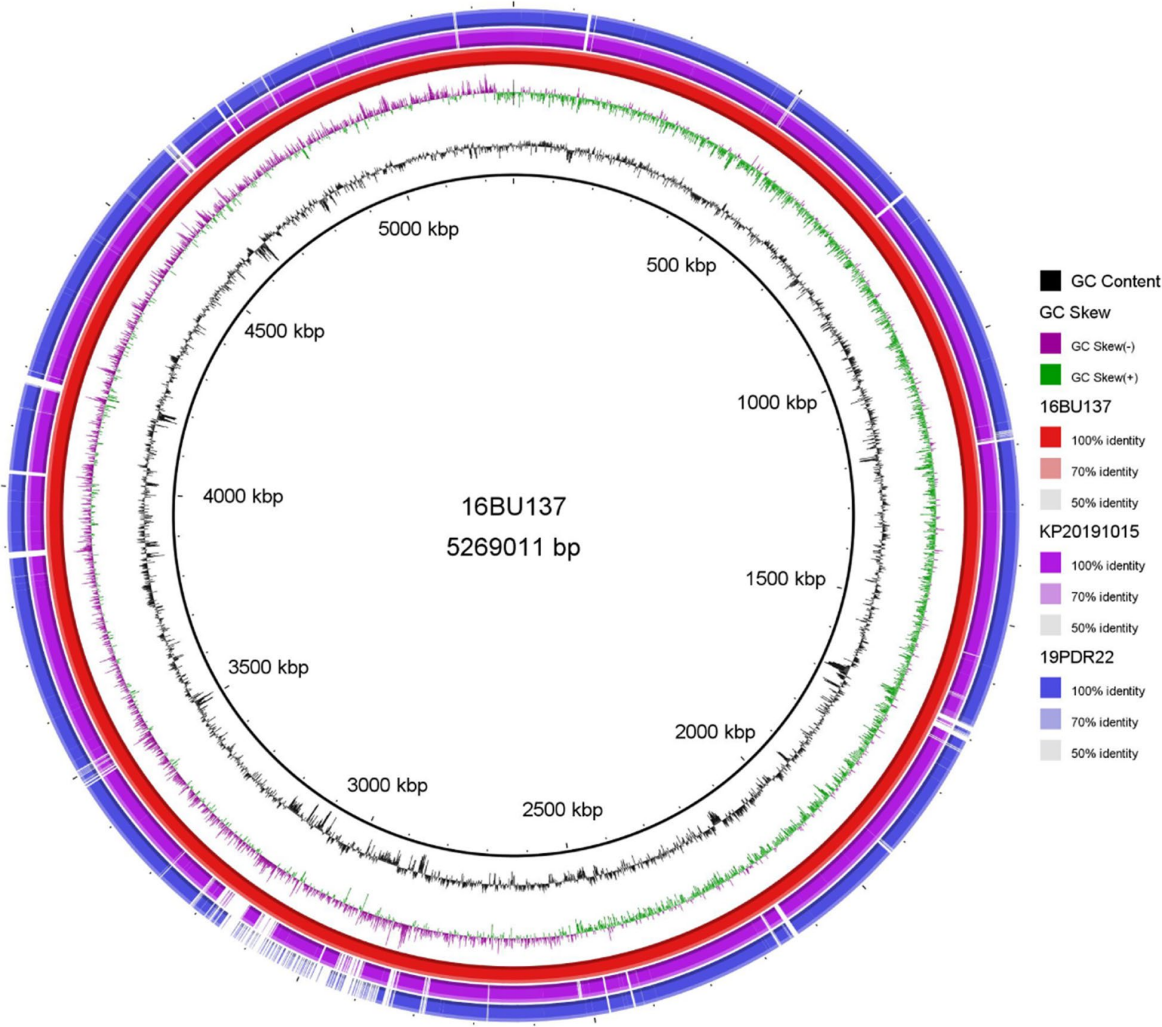
Circular chromosome map of *K. pneumoniae* 16BU137, *K. pneumoniae* KP20191015, and *K. pneumoniae* 19PDR22. 16BU137 (accession no. CP051161), KP20191015 (accession no. CP051160), 19PDR22 (accession no. CP051159). The map was drawn using BLAST Ring Image Generator (BRIG) (http://sourceforge.net/projects/brig/)

According to third-generation whole genome sequencing, the complete genome sequence of *E. coli* 17MR471 is 4,765,524 bp, containing 4433 CDS and 87 tRNA; the genome of *K. pneumoniae* 16BU137 is 5,269,011 bp, containing 4863 CDS and 86 tRNA; the genome of *K. pneumoniae* KP20191015 is 5,409,809 bp, containing 5110 CDS and 88 tRNA; the genome of *K. pneumoniae* 19PDR22 is 5,396,045 bp, containing 5077 CDS and 87 tRNA (Fig. 2). *E. coli* 17MR471 belongs to ST1437, harboring colistin resistance gene *mcr-1.1* and other seven ARGs. *K. pneumoniae* 16BU137 belongs to ST37, harboring *mcr-1.1* and other 25 ARGs *K. pneumoniae* KP20191015 belongs to ST340, harboring a *mcr-8* variant (1698 bp, 99.71% nucleotide identity to *mcr-8*) and other 28 ARGs. *K. pneumoniae* 19PDR22 belongs to ST11, while lacked known plasmid-mediated colistin resistance gene.

### Molecular epidemiological features of *mcr*-positive *E. coli* and *K. pneumoniae* isolates

**Fig. 3:**
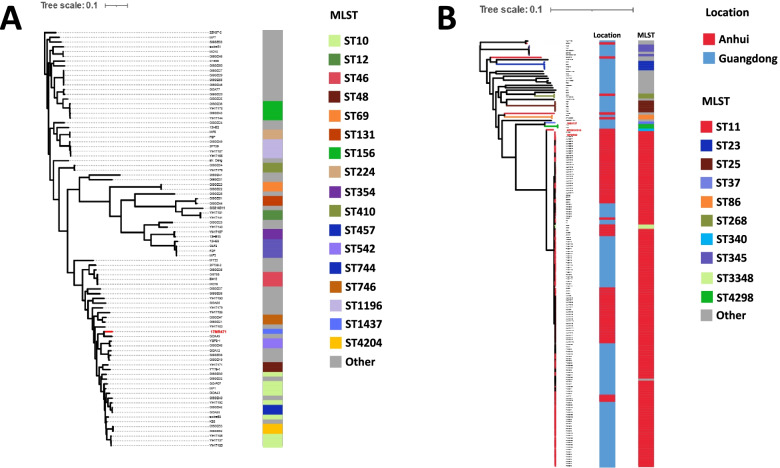
Phylogenetic analysis of *E. coli* and *K. pneumoniae* isolates. **A** Phylogenetic analysis of *E. coli* isolates in Guangdong, China. Isolates obtained in this study are highlighted in red. **B** Phylogenetic analysis of *K. pneumoniae* isolates in Anhui and Guangdong, China. The analysis was performed using Parsnp [54] and iTOLv4 [55]

To better understand the genetic background of these colistin-resistant strains, we collected all *E. coli* isolates from Guangdong, China and *K. pneumoniae* isolates from Anhui and Guangdong, China in the NCBI database, and conducted a phylogenetic analysis on them (Fig. 3). The MLST type of *E. coli* in Guangdong shows diversified characteristics. The MLST type of *K. pneumoniae* in Anhui and Guangdong is more concentrated, most of which are ST11. Among the isolates we obtained, except for KP20191015, none of the other isolates formed an independent branch. 17MR471 formed a branch with a ST6335 *E. coli* isolate GDA49. 16BU137 formed a branch with *K. pneumoniae* P10 and P12 isolates of MLST type ST4298 from Guangdong. 19PDR22 was clustered with ST11 type *K. pneumoniae* isolates. It is worth noting that KP20191015 formed a branch on its own. The *K. pneumoniae* isolates distributed in Anhui and Guangdong were intertwined in the phylogenetic tree, which seems to indicate that the *K. pneumoniae* in China has spread and needs to be controlled immediately.

### Virulence factors and colistin-related resistance genes of four isolates

A total of 48 virulence factors were predicted in *E. coli* 17MR471, 16 virulence factors were predicted in *K. pneumoniae* 16BU137, 10 virulence factors were predicted in *K. pneumoniae* KP20191015, 25 virulence factors were predicted in *K. pneumoniae* 19PDR22 (Table 3 and Table S1). In *E. coli* 17MR471, *K. pneumoniae* KP20191015 and *K. pneumoniae* 19PDR22, the identified virulence factors were all located on the chromosomes. In *K. pneumoniae* 16BU137, a total of 6 virulence factors located on the plasmid were identified. *iucA*, *iucB*, *iucC*, *iutA* and *cseA* are located on IncFIB(K)/IncFII type plasmids, while *astA* is located on IncQ1/IncFII type plasmid.

**Table 3:** Virulence factors predicted with VFDB database

Gene	16BU137	17MR471	19PDR22	KP20191015
*aslA*	–^*a*^	+ ^*b*^	–	–
*astA*	+	+	–	–
*cseA*	+	+	–	–
*csgB/F/G*	–	+	–	–
*ecpA/B/C/D/E/R*	+	+	+	+
*entA/B*	+	+	+	+
*entC/D/E/F/S*	–	+	–	–
*espL1*	–	+	–	–
*espR1*	–	+	–	–
*espX1*	–	+	–	–
*espX4*	–	+	–	–
*fepA*	–	+	–	–
*fepB*	–	+	–	–
*fepC*	+	+	+	+
*fepD*	–	+	–	–
*fepG*	–	+	–	–
*fes*	–	+	–	–
*fimA/B/C/D/E/F/G/H/I*	–	+	–	–
*fyuA*	–	–	+	–
*gspD/E/F/G/H/I*	–	+	–	–
*gspK/L/M*	–	+	–	–
*irp1/2*	–	–	+	–
*iucA/B/C*	+	–	+	–
*iutA*	+	–	+	–
*ompA*	+	+	+	+
*rmpA2*	–	–	+	–
*ybtA/E/P/Q/S/T/U/X*	–	–	+	–

^*a*^–, indicates virulence factor negative

^*b*^+, indicates virulence factor positive

Colistin resistance related genes and other resistance genes in four isolates are shown in Tables 4 and 5 and Table S2. Colistin resistance related genes in *K. pneumoniae* KP20191015 are similar to *K. pneumoniae* 19PDR22. Compared with *K. pneumoniae* 16BU137, *K. pneumoniae* KP20191015 and *K. pneumoniae* 19PDR22 may contain more colistin resistance related genes. Among the three strains, the types of *arnD*, *eptB*, *mgrB*, *opgE*, *pmrA*, *pmrB*, *pmrC*, and *pmrD* are the same. Both *K. pneumoniae* KP20191015 and *K. pneumoniae* 19PDR22 contain two types of *emrA*, two types of *emrB*, and three types of *phoP*. *K. pneumoniae* 16BU137 contains one type of *emrA*, one type of *emrB*, and two types of *phoP*. In addition, IS*903* inserted on the upstream sequence of *mgrB* in *K. pneumoniae* 19PDR22 (Fig. 1B), which may affect the normal expression of *mgrB* [56].

**Table 4:** Colistin resistance related gene in four isolates

Gene	16BU137	17MR471	19PDR22	KP20191015
*arnD*	Uniprot ID: P76472	Uniprot ID: P76472	Uniprot ID: P76472	Uniprot ID: P76472
*emrA*	Uniprot ID: P27303	Uniprot ID: P27303	Uniprot ID: P0DPR6*2; Uniprot ID: P27303	Uniprot ID: P0DPR6*2; Uniprot ID: P27303
*emrB*	Uniprot ID: P0AEJ0	Uniprot ID: P0AEJ0	Uniprot ID: P0DPR7; Uniprot ID: P0AEJ0	Uniprot ID: P0DPR7; Uniprot ID: P0AEJ0
*eptB*	Uniprot ID: P37661	Uniprot ID: P37661	Uniprot ID: P37661	Uniprot ID: P37661
*mcr-like genes*	*mcr-1.1*	*mcr-1.1*	None	*mcr-8*
*mgrB*	Uniprot ID: B5XQ45	Uniprot ID: P64512	Uniprot ID: B5XQ45, *IS 903* is inserted upstream	Uniprot ID: B5XQ45
*opgE*	Uniprot ID: P75785	Uniprot ID: P75785*2	Uniprot ID: P75785	Uniprot ID: P75785
*phoP*	Uniprot ID: P13792; Uniprot ID: D0ZV90	Uniprot ID: P23836	Uniprot ID: P0DM78; Uniprot ID: P13792; Uniprot ID: D0ZV90	Uniprot ID: P0DM78; Uniprot ID: P13792; Uniprot ID: D0ZV90
*phoQ*	Uniprot ID: P23837	Uniprot ID: P23837	Uniprot ID: P23837	Uniprot ID: P23837
*pmrA* (*basR*)	Uniprot ID: P30843	Uniprot ID: P30843	Uniprot ID: P30843	Uniprot ID: P30843
*pmrB* (*basS*)	Uniprot ID: P30844	Uniprot ID: P30844	Uniprot ID: P30844	Uniprot ID: P30844
*pmrC* (*eptA*)	Uniprot ID: P36555	Uniprot ID: P30845	Uniprot ID: P36555	Uniprot ID: P36555
*pmrD*	Uniprot ID: P37589	Uniprot ID: P37590	Uniprot ID: P37589	Uniprot ID: P37589

**Table 5:** Antimicrobial resistance genes predicted with ResFinder-3.2

Gene	Identity(%)	Antibiotic_Resistance	Position
KP20191015			
*aac(3)-IV*	100	Aminoglycoside	Plasmid
*aadA1*	100	Aminoglycoside	Plasmid
*aadA2b*	99.87	Aminoglycoside	Plasmid
*aph(3″)-Ib*	100	Aminoglycoside	Plasmid
*aph(3′)-Ia*	100	Aminoglycoside	Chromosome/Plasmid
*aph(6)-Id*	100	Aminoglycoside	Plasmid
*armA*	100	Aminoglycoside	Plasmid
*blaCTX-M-15*	100	Beta-lactam	Plasmid
*blaDHA-1*	100	Beta-lactam	Plasmid
*blaSHV-182*	99.88	Beta-lactam	Chromosome
*blaTEM-1B*	100	Beta-lactam	Plasmid
*mcr-8*	99.71	Colistin	Plasmid
*fosA*	99.27	Fosfomycin	Plasmid
*mph(A)*	100	Macrolide	Plasmid
*mph(E)*	100	Macrolide	Plasmid
*msr(E)*	100	Macrolide	Plasmid
*catA2*	96.11	Phenicol	Plasmid
*cmlA1*	99.92	Phenicol	Plasmid
*oqxA*	100	Quinolone	Chromosome
*oqxB*	100	Quinolone	Chromosome
*qnrB4*	100	Quinolone	Plasmid
*sul1*	100	Sulphonamide	Plasmid
*sul3*	100	Sulphonamide	Plasmid
*tet(D)*	100	Tetracycline	Plasmid
19PDR22			
*aac(3)-IId*	99.88	Aminoglycoside	Chromosome
*aadA2b*	99.87	Aminoglycoside	Plasmid
*aadA5*	100	Aminoglycoside	Plasmid
*aph(3″)-Ib*	100	Aminoglycoside	Plasmid
*aph(6)-Id*	100	Aminoglycoside	Plasmid
*armA*	100	Aminoglycoside	Plasmid
*rmtB*	100	Aminoglycoside	Plasmid
*blaCTX-M-65*	100	Beta-lactam	Plasmid
*blaKPC-2*	100	Beta-lactam	Plasmid
*blaSHV-12*	100	Beta-lactam	Chromosome/Plasmid
*blaSHV-182*	99.77	Beta-lactam	Chromosome
*blaTEM-1A*	100	Beta-lactam	Plasmid
*blaTEM-1B*	100	Beta-lactam	Chromosome/Plasmid
*blaTEM-1C*	100	Beta-lactam	Plasmid
*fosA*	99.27	Fosfomycin	Chromosome
*mph(A)*	100	Macrolide	Chromosome
*mph(E)*	100	Macrolide	Plasmid
*msr(E)*	100	Macrolide	Plasmid
*sul1*	100	Sulphonamide	Plasmid
*sul2*	100	Sulphonamide	Plasmid
*dfrA17*	100	Trimethoprim	Plasmid
16BU137			
*aac(3)-IId*	99.88	Aminoglycoside	Plasmid
*aac(6′)-Ib-cr*	100	Aminoglycoside	Plasmid
*aadA16*	99.65	Aminoglycoside	Plasmid
*aph(3″)-Ib*	100	Aminoglycoside	Plasmid
*aph(3′)-Ia*	99.88	Aminoglycoside	Plasmid
*aph(6)-Id*	100	Aminoglycoside	Plasmid
*blaCTX-M-3*	100	Beta-lactam	Plasmid
*blaSHV-110*	99.77	Beta-lactam	Chromosome
*blaTEM-1B*	100	Beta-lactam	Plasmid
*mcr-1.1*	100	Colistin	Plasmid
*fosA*	99.29	Fosfomycin	Chromosome
*mph(A)*	100	Macrolide	Plasmid
*floR*	98.27	Phenicol	Plasmid
*aac(6′)-Ib-cr*	100	Quinolone	Plasmid
*oqxA*	100	Quinolone	Chromosome
*oqxB*	100	Quinolone	Chromosome
*qnrB2*	99.84	Quinolone	Plasmid
*qnrS1*	100	Quinolone	Plasmid
*ARR-3*	100	Quinolone	Plasmid
*sul1*	100	Rifampicin	Plasmid
*sul2*	99.88	Sulphonamide	Plasmid
*tet(A)*	100	Tetracycline	Plasmid
*dfrA27*	100	Trimethoprim	Plasmid
17MR471			
*blaTEM-1B*	100	Beta-lactam	Plasmid
*mcr-1.1*	100	Colistin	Chromosome
*mdf(A)*	99.92	Macrolide	Chromosome
*floR*	98.19	Phenicol	Plasmid
*oqxA*	100	Quinolone	Plasmid
*oqxB*	99.97	Quinolone	Plasmid
*tet(B)*	100	Tetracycline	Chromosome
*tet(M)*	96.15	Tetracycline	Plasmid

### Location of *mcr-1.1* on chromosome and plasmid

**Fig. 4:**
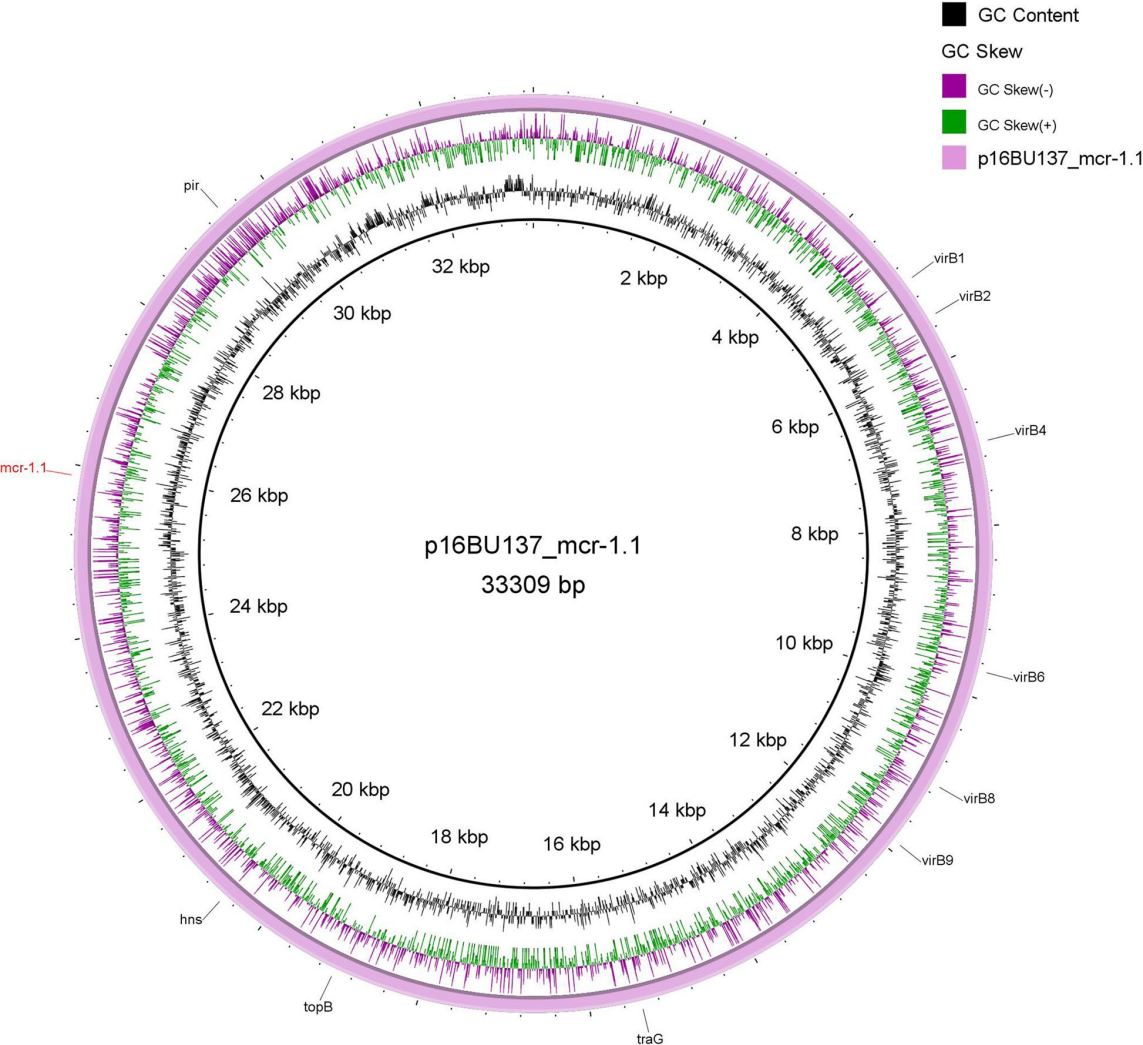
Circular chromosome map of p16BU137_mcr-1.1

The *mcr-1.1* gene was found to locate on the chromosome of *E. coli* 17MR471. Specifically, the *mcr-1*-*pap2* gene cassette which encodes both MCR-1 and a hypothetical protein was flanked by two copies of IS*Apl1* (1070 bp, IS30 family) upstream and downstream in the same orientation. In *K. pneumoniae* 16BU137, *mcr-1.1* located in an IncX4-type plasmid which named p16BU137_mcr-1.1 (Table S3). This plasmid is 33,309 bp in size and is predicted to encode 41 ORFs for which *mcr-1.1* is the only resistance gene. No IS*Apl1* was found in the flanking sequences of *mcr-1.1* in p16BU137_mcr-1.1(Fig. 4). IncX4 is the dominant plasmid type to harbor *mcr-1.1* [57]. The *mcr*-1-bearing IncX4 plasmid was firstly identified in Germany in 2009. Since 2009, the majority of *mcr-1* genes have been found on IncX4 plasmids. BLASTn revealed that the genetic context of *mcr-1.1* in IncX4 plasmids are diverse. The examples included that *mcr-1.1* without flanking IS*Apl1* (pAF48, KX032520). Also, *mcr-1-pap2* could be flanked by IS*Apl1* upstream (pMCR-11EC-P293, KX555451), downstream (pPY1, KX711708) or both (pC214, KY120363). Plasmids like PN42 (MG557854) and pCDF8 (MF175191) have truncated IS elements in flanking regions of *mcr-1*. It has been hypothesized that after the loss of IS*Apl1*, *mcr-1* is immobilized in the plasmids [18].

### A *mcr-8* variant was found in an IncFIB/ IncFII plasmid

**Fig. 5:**
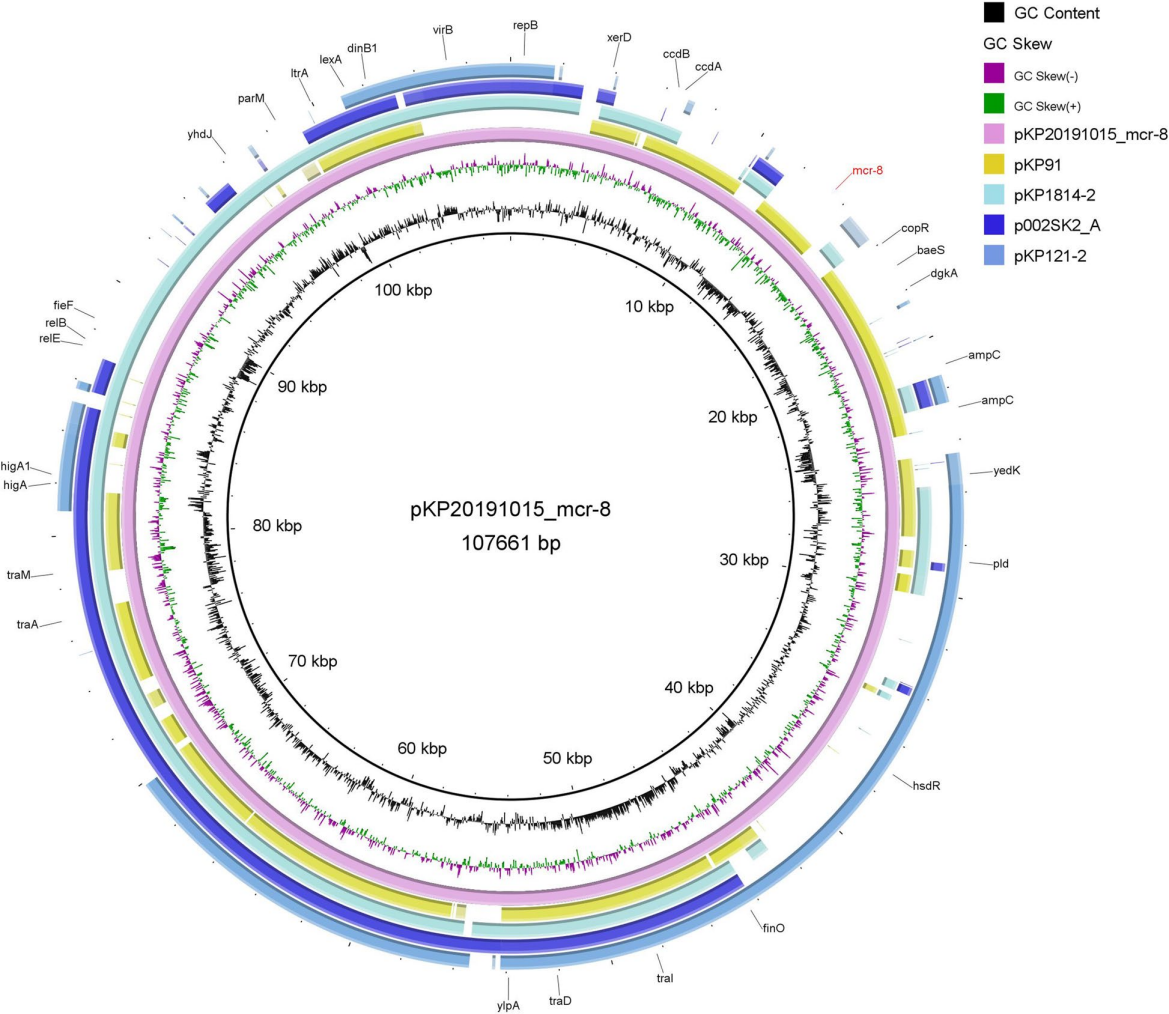
Schematic presentation of major structural features of pKP20191015_mcr-8 in comparison with the reference plasmids pKP91, pKP1814–2, p002SK2_A, and pKP121–2. pKP20191015_mcr-8 (accession no. MT316509), pKP91 (accession no. MG736312), pKP1814–2 (accession no. KX839208), p002SK2_A (accession no. CP025516), pKP121–2 (accession no. CP031851). Annotation features represented the genes in pKP20191015_mcr-8

In *K. pneumoniae* KP20191015, a *mcr-8* variant was found in an 107,661 bp IncFIB/ IncFII plasmid which named pKP20191015_mcr-8. *mcr-8* was flanked by a reverse IS*Ecl1*-like element (1336 bp, 99% identity to IS*Ecl1*) upstream. Also, it was flanked by an IS*Kpn26*-like element (1196 bp, 99% similarity to IS*Kpn26*) at the same direction downstream. Consistent with the sequences in the *mcr-8*-carrying pKP91 (95,983 bp, MG736312) [48], both of which carried *dgkA*, *baeS*, and *copR* close to *mcr-8* (Fig. 5). While *mcr-8* in pKP91 was flanked by two intact IS*903B* sequences up- and downstream [48], and significant differences were observed in the remaining plasmid backbone (Fig. 5). BLASTn indicated that pKP20191015_mcr-8 carried novel components that showed limited identity to those known plasmid sequences (coverage < 75%). pKP20191015_mcr-8 is organized similar to that of plasmid pKP1814–2 (187,349 bp, KX839208) (69% coverage, 99.84% identity) identified in *K. pneumoniae* in China; p002SK2_A (159,714 bp, CP025516) (53% coverage, 99.80% identity) identified in *K. pneumoniae* in Switzerland; pKP121–2 (134,208 bp, CP031851) (53% coverage, 99.75% identity) identified in *K. pneumoniae* in China. They all carried plasmid transfer associated *tra* locus with different combination and the replicon encoding gene *repB*.

## Discussion

Polymyxins are cyclic, positively charged peptides, which were first discovered to possess antibiotic properties in the 1940s [58]. Polymyxins can bind to lipid A of lipopolysaccharide (LPS) on the outer membrane of Gram-negative bacteria, and then displace Mg^2+^ and Ca^2+^ from cationic binding sites leading to disruption of bacterial membrane integrity [58, 59]. Polymyxins (polymyxin B and colistin) are a last resort treatment against human infections caused by multidrug-resistant (MDR) Gram-negative bacteria [60]. Colistin resistance is often associated with chromosomal point mutations that affect the expression of regulators, which modify lipid A and lead to alterations of LPS [61]. Bacteria can add phosphoethanolamine (PEtN) and 4-amino-4-deoxy-L-arabinose (L-Ara4N) to lipid A via biosynthesis, thereby decreasing the net negative charge of lipid A to reduce its binding affinity to polymyxins [62, 63]. The synthesis and transfer of PEtN and L-Ara4N are mediated by the expression of *pmrCAB* and *arnBCADTEF* (also called *pmrHFIJKLM*) [64] which were regulated by a two-component system (TCS) PmrA/PmrB [65, 66]. Mutations in the genes encoding PmrA/PmrB were shown to contribute to polymyxin resistance [67, 68]. Moreover, another TCS PhoP/PhoQ is known to develop polymyxin resistance via activation of its posttranscriptional activator PmrD to induce expression of the PmrA/PmrB system [69]. Mutations in the genes encoding the PhoP/PhoQ were also associated with colistin resistance [70]. Here, we report four polymyxin-resistant *Enterobacteriaceae* strains. Among them, *K. pneumoniae* 16BU137 and *E. coli* 17MR471 carries *mcr-1*, and *K. pneumoniae* KP20191015 carry *mcr-8*. The *mcr-1* in 17MR471 is located on the chromosome, and the surrounding sequence is a typical *Tn 6330* structure, (IS*Apl1*)2-*mcr-1*-*pap*2. The *mcr-1* in 16BU137 lacks upstream IS*Apl1* and downstream IS*Apl1*, but still retains *pap*2. IS*Apl1* may be lost due to its involvement in *mcr-1* transposition [18, 19]. However, *pap2* always exists downstream of *mcr-1*, which seems to suggest that *pap*2 may play an indispensable function for *mcr-1*. Through further experimental verification, we identified *K. pneumoniae* 19PDR22 which conferred high MIC of colistin, while no known plasmid-mediated colistin genes was found. We found an IS*903B*-like element (97% similarity to IS*903B*) inserted into the upstream sequence of *mgrB*. This insertion appeared at position − 18 bp of the *mgrB*, which may lead to the inactivation of *mgrB* by interrupting its promoter region. The inactivation of *mgrB* conferred colistin resistance has been reported previously [71]. IS integration has also been reported to induce colistin resistance via transposition into the upstream putative promoter region of *mgrB* [72]. IS*903*, a member of IS*5* family, is implicated in antibiotic resistance. Insertion sequences of the IS*5* family have also been reported to truncate *mgrB* in *Klebsiella oxytoca* and yield elevated MICs for colistin [73]. More studies are needed to evaluate the mobilization of these elements from plasmids to the chromosome to disrupt the expression of potential resistance-associated genes. Our data show that the transformation efficiency of p16BU137_mcr-1.1 is higher than that of pKP20191015_mcr-8. This may indicate that the plasmid carrying *mcr-1* has a higher transformation efficiency and stronger transmission ability than the plasmid carrying *mcr-8*. However, the experimental results still have limitations due to the small number of strains in this study. 16BU137 and KP20191015 carries the *mcr*-like genes, meanwhile carries a variety of ESBL genes, such as *bla*_SHV_, *bla*_CTX_ and *bla*_TEM_. The existence of these resistance genes makes MDR enterobacteria a huge threat to public medical and health safety.

## Conclusion

We present the complete genome of four polymyxin-resistant strains (including two clinically isolated strains and two environmentally isolated strains, both clinically isolated strains are *K. pneumoniae*). The high-quality complete genome sequence generated in this study will help to further study the mechanism of polymyxin resistance of *K. pneumoniae* and the horizontal transfer pathway of *mcr*-like genes. Although two strains are isolated from the environment, they still have high polymyxin resistance. And the types of virulence factors are basically the same as clinical strains, and still have the risk of infecting humans. These also warns us that the multi-drug resistant *K. pneumoniae* has spread seriously in China and needs to be controlled as soon as possible.

## Methods

### Bacterial isolation

The MIC of polymyxin B was tested on the MDR clinical isolates isolated from the inpatients in Affiliated Hospital of Anhui University of Traditional Chinese Medicine and the Anhui Provincial Hospital in 2019, and two polymyxin B resistant isolates were obtained (*K. pneumoniae* 19PDR22 and *K. pneumoniae* KP20191015). The environmental isolates of *K. pneumoniae* 16BU137 and *E. coli* 17MR471 were obtained from our previous studies [32]. They all carried *mcr-1* and were resistant to polymyxin B. Briefly, the environmental samples were collected using sterilized swab with saline, and cultured by broth medium. Then, the cultured samples were plated on the MacConkey agar with colistin (2 μg/mL) and cultured under 37 °C overnight. Subsequently, we randomly selected 5 colonies for each plate which were subject to screen *mcr-1* gene by PCR. Only one colony for each sample was included for the subsequent study. *K. pneumoniae* 19PDR22 was isolated from the urine of patient with urinary tract infection, and *K. pneumoniae* KP20191015 was isolated from the sputum of patient with severe pneumonia. Sputum and urine were plated on blood agar plates and cultured at 37 °C to isolate bacterial clones. VITEK 2 Compact System (bioMérieux, France) was used to identify positive culture strains.

### Determination of minimum inhibitory concentration

*K. pneumoniae and E. coli* were cultured overnight in LB liquid medium at 37 °C for 220 rpm according to 1:100, and a small amount of liquid medium was streaked on LB plate and incubated overnight in 37 °C constant temperature incubator. Several monoclonal strains were selected to adjust the concentration of bacteria in MH (Mueller-Hinton Broth) medium so that the concentration of bacteria reached OD_600_ = 0.4 and then diluted 200 times in MH medium [74]. Mix 75 ml of MH medium with different concentrations of polymyxin B and 75 ml of MH medium with diluted bacterial solution and add them to each well of a 96-well plate according to the polymyxin concentration gradient. The final CFU of the well is 5 × 10^5^. Each concentration gradient was divided into three parallel groups and grown at 37 °C and 220 rpm with shaking for 24 and 48 h. The experiment was repeated three times independently.

### Plasmid conjugation experiments

*E. coli* J53 (LacZ–, AzrR, RifR) was used as the recipient, and the *mcr*-like gene-positive strain (16BU137, KP20191015) was used as the donor. Overnight culture (2 mL) of each donor and recipient bacteria was mixed together at a ratio of donor to recipient of 1:3. The mixture was added to a final volume of 5 mL LB liquid medium, and incubate at 37 °C for 12–18 h. Then spotted the mixture on Muller-Hinton agar plates containing 100 mg/L sodium azide and 2 mg/L polymyxin B as a selective medium for *E. coli* J53 transconjugants. Detection of *mcr*-like gene by PCR confirmed the putative transconjugants. Use *wzi* gene primers, *mcr-1* gene primers and *mcr-8* gene primers to distinguish the recipient strain (16BU137 and KP20191015) from the donor strain (J53).

### Whole-genome sequencing and genotyping

*K. pneumoniae* and *E. coli* were cultured overnight in LB medium. Bacterial samples (5000 g 10 min at 4 °C) were collected and frozen at − 80 °C. The genomes of four isolates were performed using a PacBio RS II platform and Illumina HiSeq 4000 platform at the Beijing Genomics Institute (BGI, Shenzhen, China). Four SMRT cells Zero-Mode Waveguide arrays of sequencing, were used by the PacBio platform to generate the subreads set. PacBio subreads (length < 1 kb) were removed. The program Pbdagcon (https://github.com/PacificBiosciences/pbdagcon) was used for self-correction. Draft genomic unitigs, which are uncontested groups of fragments, were assembled using the Celera Assembler against a high quality corrected circular consensus sequence subreads set. To improve the accuracy of the genome sequences, GATK (https://www.broadinstitute.org/gatk/) and SOAP tool packages (SOAP2, SOAPsnp, SOAPindel) were used to make single-base corrections.

De novo hybrid assembly both of short Illumina reads and long PacBio reads was performed using Unicycler v0.4.3 [75]. Complete circular contigs were then corrected using Pilon v1.22 with Illumina reads. For each de novo assembled genome, coding sequences were predicted using Prodigal (v. 2.6) [76] and annotated using the rapid prokaryotic genome annotation tool Prokka [77]. Acquired antimicrobial resistance genes (ARGs) were identified using ABRicate version 0.5 (https://github.com/tseemann/abricate) by aligning genome sequences to the ResFinder database [78]. The virulence factors of the isolates were identified using VFDB database [79]. Insertion sequence (IS) elements were determined with ISFinder (https://www-isfinder.biotoul.fr). In silico multilocus sequence typing (MLST) was performed by MLST 1.8 (https://cge.cbs.dtu.dk/services/MLST/). Plasmid replicon types were detected using PlasmidFinder v1.3 [80].

### Phylogenetic analysis

We collected all 87 *E. coli* strains from Guangdong, China and all 182 *K. pneumoniae* strains from Guangdong and Anhui, China (182 strains from Guangdong and 70 from Anhui) in the NCBI database (https://www.ncbi.nlm.nih.gov/pathogens/) as of December 2020. HarvestTools kit (Parsnp, Gingr and HarvestTools) was used to perform comparative genomics analysis and phylogenetic analysis of different isolates, Interactive tree of life (iTOL) v5 (http://itol.embl.de/) was used to construct a maximum likelihood phylogenetic tree [54, 55].

## Supplementary Information

**Additional file 1.**

**Additional file 2.**

**Additional file 3.**

## Data Availability

Nucleotide sequence accession number whole-genome sequencing data have been deposited in the NCBI database and are publicly available under BioProject: PRJNA622869. The complete genome sequence of *E. coli* 17MR471, *K. pneumoniae* 16BU137, *K. pneumoniae* KP20191015, and *K. pneumoniae* 19PDR22 reported in this study has been submitted to the NCBI database and assigned accession number CP051158, CP051161, CP051160, and CP051159, respectively. Sequences of p16BU137_mcr-1.1 and pKP20191015_mcr-8 were assigned accession number MT316509 and MT316510, respectively. The detailed prediction information of virulence factors, resistance genes and plasmids are located in Table S1, Table S2 and Table S3, respectively.

## Abbreviations

MDR: Multidrug resistant
CPS: Capsular polysaccharide
NDM: New Delhi β-lactamase
ESBL: Extended spectrum β-lactamase genes
PEA: Phosphoethanolamine
LPS: Lipopolysaccharide
TSDs: Target site duplications
WGS: Whole-genome sequencing
PEtN: Phosphoethanolamine
